# Cadmium availability in rhizosphere and non-rhizosphere soils in cacao farms in Santander, Colombia

**DOI:** 10.1007/s10661-024-13301-x

**Published:** 2024-11-26

**Authors:** C. A. Adarme-Duran, J. Ágreda, P. F. B. Brandão, E. Castillo

**Affiliations:** 1 Universidad Nacional de Colombia, sede Bogotá, Facultad de Ciencias, Instituto de Biotecnología, Carrera 30 # 45-03, Bogotá, Colombia; 2 Universidad Nacional de Colombia, sede Bogotá, Facultad de Ciencias, Departamento de Química, Grupo de Estudios para la Remediación y Mitigación de Impactos Negativos al Ambiente (GERMINA), Carrera 30 # 45-03, Bogotá, Colombia

**Keywords:** Available Cd, Cocoa, Rhizosphere soils, Bulk soils, Soil parameters, Spatial distribution

## Abstract

Current research has highlighted the need to understand the factors influencing cadmium (Cd) availability in cacao-growing soils to elucidate its presence in cacao beans (the raw material for chocolate). Although literature about this topic is increasing, few report the importance of rhizosphere soils on Cd dynamics. This study aimed to understand the changes in available Cd and its association with soil properties (pH, pseudo-total Cd, available Cd (Cd-DTPA), Ca, Mg, K, Na, soil organic carbon, P, Zn, urease activity, exchangeable acidity, and cation exchange capacity) considering rhizosphere and non-rhizosphere soils. Both soil types (51 samples of each, 102 in total) were collected from two Colombian cacao farms. The medians of pseudo-total Cd (1.86 mg kg^−1^) and Cd-DTPA (0.76 mg kg^−1^) were, respectively, about threefold and fourfold higher in rhizosphere compared to non-rhizosphere soils. Principal component analysis showed a clear distinction between rhizosphere and non-rhizosphere soils based on differences in soil properties, which explained the observed changes in available Cd when comparing both soil types. Soil organic carbon and Zn were important drivers of available Cd in rhizosphere soils. Spatial distribution analysis revealed a tendency of available Cd to cluster in rhizosphere soils, and indicated hotspots within each farm. These findings highlight the importance of rhizosphere soils for Cd diagnosis and monitoring, and for improving knowledge about Cd dynamics in the soil-*Theobroma cacao* L. system.

**Supplementary Information:**

The online version contains supplementary material available at 10.1007/s10661-024-13301-x.

## Introduction

The Colombian cacao sector represents an important socio-economic activity with more than 45.000 producers, who are small farmerholders with an average of 2.66 hectares planted with cacao (FEDECACAO, 2024). This crop supports the consolidation of peace in the country, as an alternative to illicit coca cultivation (Abbott et al., 2018). Eighty percent of the country's cacao exports are classified as fine flavor cacao, which is granted to only 12% of cacao beans exported worldwide (ICCO, 2024). However, this select market is threatened by the presence of cadmium (Cd) in cacao beans affecting not only Colombia but other countries in Latin America and the Caribbean (Meter et al., 2019). Cadmium is a metal classified as a Group 1 Human Carcinogen (IARC, 2012) that can be found in agricultural soils and is easily transferred to crops, thus entering the food chain (Kubier et al., 2019).

Cadmium in cacao beans is related to its presence in soil and to soil physicochemical properties (Vanderschueren et al., 2021). Thus, the analysis of soil Cd concentration, the available fraction of Cd in soil, and its associations with soil physicochemical properties have been crucial. Cadmium contamination has been reported in the cacao agricultural system in Africa, Asia, Central America, and South America (Carrillo et al., 2023; Maddela et al., 2020; Meter et al., 2019). Different pseudo-total (from 0.01 to 27.00 mg kg^−1^) and available (from < 0.05 to 7.48 mg kg^−1^) Cd concentrations have been reported in cacao farms soils (Bravo et al., 2024; Chavez et al., 2015; Gramlich et al., 2017, 2018; Rodríguez-Albarracín et al., 2019). The association between soil properties and Cd in cacao-growing soils has identified the importance of pH, organic carbon, Zn, and Ca (Bravo et al., 2021; Scaccabarozzi et al., 2020; Vanderschueren et al., 2021). Despite the advances in understanding soil Cd content and availability in cacao-growing soils, further research is needed, including other important aspects in the context of soil–plant systems.

In several studies, cacao-growing soil samples were collected from the top layer, between 0 and 30 cm, as part of vertical Cd distribution research to establish possible contamination/enrichment pathways (Bravo et al., 2021; Chavez et al., 2015; Gramlich et al., 2017, 2018; Scaccabarozzi et al., 2020). Some of the latter references argue that this soil vertical zone has active roots that participate in the metal uptake by the plant. However, they do not explicitly consider the rhizosphere zone with the soil closest to the root at a millimeter distance, where Cd availability can be influenced by plant-soil interactions (Sterckeman & Thomine., 2020).

Plants root exudates, found at distances of up to 12 mm from the root (Sauer et al., 2006), can modify soil physicochemical properties and metal availability around roots (Hinsinger et al., 2009). This fact allowed the division of soil into rhizosphere soil (RS) and non-rhizosphere soil (NRS). Root exudates can alter Cd availability by complexation, changing soil pH and Redox Potential (Eh), and altering the rhizosphere microbiota interacting with Cd in soil (Dong et al., 2007). For example, root-induced acidification has influenced Cd availability in the RS of *Sedum plumbizincicola* (Sun et al., 2019). Additionally, it has been reported that agricultural land use can change the spatial distribution of available metals (Gao et al., 2021), suggesting that soil–plant interactions could change the spatial distribution patterns of Cd in RS compared to NRS.

Despite its importance, the association between soil's chemical properties and available Cd in it has been little investigated when considering rhizosphere and non-rhizosphere soils at the field scale. This is partly because rhizosphere soil sampling has technical difficulties, specially in the case of perennial trees such as cacao, which have a complex root architecture due to their physical anchoring (Pregitzer, 2008). However, several procedures have been developed to cope with the separation of RS and NRS (Luster et al., 2009). This work aimed to assess the distribution of Cd content in RS and NRS from two cacao farms located in Santander—Colombia, and to investigate the relationship of available Cd with soil properties of both soil types. Classical statistics and principal component analysis were used to differentiate RS and NRS, and to study the changes in available Cd in the soil. The spatial distribution patterns and hotspots of available Cd in the RS and NRS are found using geostatistical techniques. These findings allow a better diagnosis of Cd in soil, and emphasize the importance of RS to understand Cd availability in cacao-growing soils.

## Materials and methods

### Site description

Samples were collected from two farms, *La Perla* (6° 39′ 49.17" N 73° 30′ 40.81" W) and *Los Cedros* (6° 39′ 26.81" N 73° 30′ 21.87" W), which have 3500 and 1400 total planted cacao trees, respectively, with an average age of 10 years. The cacao genotypes present in each farm are CCN-51, FEC2, FEC1, ICS95 and CAUCASIA for *La Perla* farm, and ICS95, ICS60, Selección Colombia, CCN-51 and FEC2, for *Los Cedros* farm. Both farms are located in *El Carmen de Chucurí* municipality, Santander department, which is the leading cacao producer in Colombia, accounting for 34% of the national production in 2023 (FEDECACAO. 2024). *El Carmen de Chucurí* has a cacao plantation area of 10.911 hectares with an average cacao yield of 453.77 kg^−1^ ha^−1^ yr^−1^ (FEDECACAO. 2024). Specifically, *La Perla* and *Los Cedros* farms have a cacao yield of 1000 kg^−1^ ha^−1^ yr^−1^ (on 2,5 hectares) and 700 kg^−1^ ha^−1^ yr^−1^ (on 1,5 hectares), respectively. The climate of this region is humid tropical according to the Köppen classification (Peláez et al., 2018), with monthly rainfall between 26 and 463 mm (July 2020—March 2021), characterized by a bimodal regimen, and an average temperature of 27 °C (IDEAM. 2022). Soil texture was mostly clay loam on both farms*.*

### Sampling strategy and pre-treatment

**Fig. 1:**
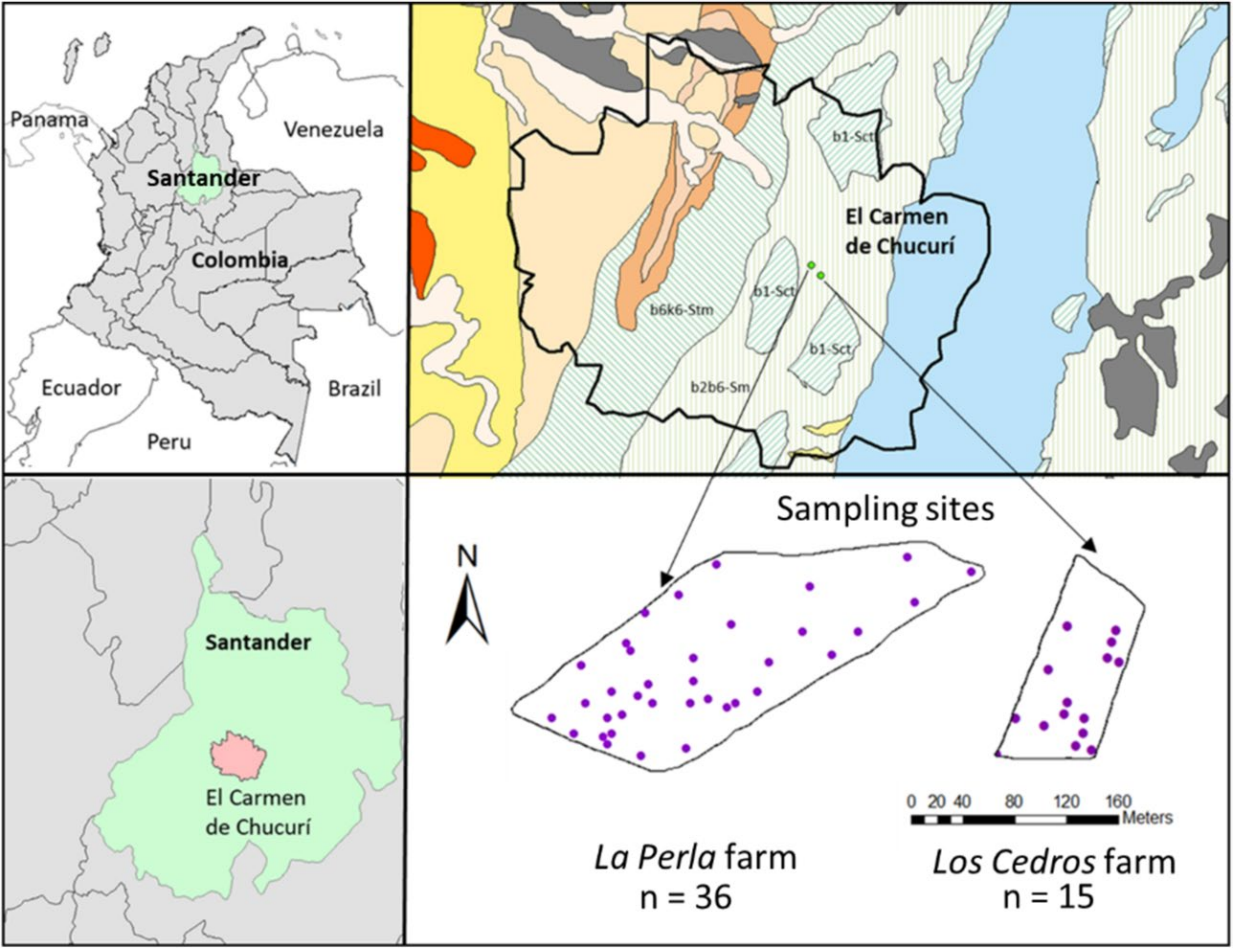
Sampling sites at *La Perla* and *Los Cedros* farms located in *El Carmen de Chucurí*, Santander, Colombia. The farms are located in the geological subunit b2b6-Sm. Adapted from Servicio Geológico Colombiano (Gómez & Montes., 2020)

The sampling was carried out in March 2021. The number of sampling sites on each farm varied according to the production area and the total number of trees on each farm (covering 1% distributed over the entire planted area). Thus, 36 and 15 sites were selected from *La Perla* and *Los Cedros* farms, respectively (Fig. 1), and 102 soil samples were collected in total: 51 rhizosphere soil (RS) samples and 51 non-rhizosphere soil (NRS) samples. Prior to soil sample collection, the leaf litter was removed by hand from the soil. Sampling of cacao rhizosphere soils is challenging because the pivoting root system can reach depths of up to 2 m (Souza et al., 2018). However, the major active root zone is found in the topsoil (Almeida & Valle, 2008; Niether et al., 2019). In this work, we used a previously published methodology based on the “pull and shake” method (Luster et al., 2009) for the sampling of RS, where it is assumed that root exudates will keep the RS adhering to root. Additionally, we considered the distance at which plant root exudates have been found (Sauer et al., 2006). The plant's roots (0–10 cm depth) and adhering soil were carefully removed to collect the RS (Supplementary Material Fig. S1). Then, this root-soil system was manually shaken to remove the loose soil. The soil that remained adhered up to 10 mm from the root was considered RS. On the other hand, the NRS was collected between 50 to 300 mm from the root (Supplementary Material Fig. S1).

Each soil sample corresponded to a composite sample obtained by mixing 3 sub-samples from around the tree trunk and under the tree canopy at a distance of 50 to 70 cm. A Global Positioning System (GPS) receiver determined all sampling site locations. Both soil types were collected using a plastic hand shovel. For chemical analysis, the samples were oven-dried at 70 °C, disaggregated, and sieved to 2 mm particle size. To determine soil urease activity, fresh soil samples were collected in sterile Falcon tubes and stored at 4 °C until processing. The samples were analyzed as soon as possible in the laboratory.

### Chemical analysis

Soil pH was measured potentiometrically in a soil/water suspension (1:1 w/v) (IGAC. 2006). Soil organic carbon (SOC) was determined by colorimetry using the Walkley–Black method (van Reeuwijk 2002). Exchangeable base cations (Ca, Mg, Na, K) were extracted using ammonium acetate (1 M, pH 7,0) and measured by Flame Atomic Absorption Spectroscopy. It has been reported that soil microorganism can alter Cd availability (Seshadri et al., 2015). Particularly, ureolytic bacteria can precipitate Cd in soil solution (Tamayo-Figueroa et al., 2019) thus, exploring the impact of urease activity on Cd-DTPA is of interest. Urease activity was asessed by incubating the soil sample with urea (720 mM) at 37 °C for 2 h. Then, the ammonium generated by the enzyme activity was extracted with a KCl (2 M)—HCl (0.01 M) solution for 30 min on a rotatory shaker (Kandeler et al., 2011), and ammonium was measured following a modified Berthelot method (Verdouw et al., 1978). Exchangeable acidity was determined in KCl 1 M by titration. Cation Exchange Capacity (CEC) was calculated by summing the charge equivalents of exchangeable base cations and acidity. Available phosphorus was determined using the Bray II method and measured colorimetrically (FAO. 2021). Available Cd^2+^ (diethylenetriaminepentaacetic acid (DTPA) extraction, Cd-DTPA) was determined in 12.5 g of soil using 25 g of a solution (pH = 7.3) containing 0.005 M DTPA, 0.01 M triethanolamine (TEA) and 0.01 M CaCl_2_, and orbital shaking for 2 h (Lindsay & Norvell., 1978). The pseudo-total Cd and Zn concentrations were obtained by digestion of 1 g of soil with 8 mL of aqua regia (HCl:HNO_3_ 3:1) heated in a graphite block digestion system (DigiPREP, SCP SCIENCE, Canada) with the following temperature program: an increase from room temperature to 120 °C in 30 min and hold at 120 °C for 120 min. Pseudo-total and available soil extractions were filtered (< 2 µm), made up to mass with deionized water (pseudo-total extraction: 30 g), and analyzed by Atomic Absorption Spectroscopy (CS-FAAS, ContrAA700 High Resolution Continuum Source, equipped with flame atomizer and graphite furnace, Analytik Jena, Berlin, Germany). The limits of quantification (LOQ) for Cd in liquid solutions were 0.01 mg L^−1^ for flame atomization and 0.001 mg L^−1^ for electrothermal atomization. The quality of the pseudo-total metal analysis was verified using the Certified Reference Material (CRM) NCS DC 73319a (2.5 ± 0.2 mg Cd g^−1^) with a recovery (± SD) of 2.58 (± 0.08) mg Cd g^−1^. Replicates were included in each batch, finding a relative percentage difference lower than 5%.

### Statistical analysis

Data analysis was performed using R version 4.2.2, RStudio 2022 version 12.0 + 353, and the packages psych version 2.2.9, stats version 4.2.2, factoextra version 1.0.7, and mdatools version 0.13.1. Descriptive statistics included mean, median, standard deviation, minimum, and maximum. RS and NRS were compared under the null hypothesis that the chemical properties of both soil types were equal. Since the distribution of the variables was non-normal according to the Shapiro-Wilks test, the Wilcoxon test was performed to compare variables with non-normal distributions, using a statistical significance < 0.05. Spearman correlations were used to test the relationships among the measured variables. Principal Component Analysis (PCA) was used to study the differentiation between RS and NRS, and to identify important variables. CEC was not included because it is a combination of other variables (Jolliffe., 1972), nor was Na since a relevant percentage (11%) of values were reported as below the quantification limit, and data imputation is not fully effective in environmental analysis (Aruga., 1997). The data used for the PCA were standardized (centered and scaled) to avoid the influence of unit differences among the variables. Also, the outliers were identified and removed according to score and orthogonal distances (Kucheryavskiy., 2020).

### Geostatistical analysis

The spatial distribution pattern of Cd in RS and NRS was analyzed. The Global Moran Index was calculated to measure the tendency of Cd to cluster or not in space, and the Local Moran Index served to identify hot and cold spots; both were performed on ArcMap 10.8 (ESRI Inc. USA) using the Geostatistical Analyst extension. The parameters analyzed were available Cd (Cd-DTPA), Zn, and SOC. Distance band was analyzed to determine spatial autocorrelation strength (Zhang et al., 2008) based on the Z-score, where the analyzed distance range ensures that every point has at least one neighborhood and a total of n-1 neighborhoods. The statistical significance of spatial autocorrelation, Z-score, and p-value were calculated under the null hypothesis that the analyzed attribute is randomly distributed. Significant autocorrelation exists if the Z-score exceeds 1.96 and the p-value is < 0.05 (Xiao et al., 2018). Since Moran's index is affected by the distance band, the global Moran index was calculated for different distances, looking for the maximum value of the global Moran's index indicating the strongest spatial autocorrelation (Huo et al., 2012). The strongest Moran's index was selected for the variables evaluated by correlograms.

## Results and discussion

Here we present the first study on Cd content and availability in cacao-growing soils in rhizosphere (RS) and non-rhizosphere (NRS) soils at field scale in Santander, Colombia. The medians of the pseudo-total and available (DTPA-Cd) Cd concentration, and soil chemical properties with their coefficient of variation (CV, standard deviation over means) are presented in Table 1. The data show a skewed and non-normal distribution (Supplementary Material Tables S1 and S2); the CVs show high variability in most parameters. According to the Wilcoxon test, 12 of the 13 variables showed different distributions when comparing RS and NRS (p-value < 0.05). Only Zn presented a non-significant difference for both farms, and CEC was not significantly different for *Los Cedros* but significant for *La Perla* (Table 1). These results suggest different chemical environments when comparing NRS and RS, and establish a baseline for exploring a possible rhizospheric effect of cacao on soil Cd availability.

**Table 1:** Medians and coefficient of variation (within parentheses) of the variables studied in this work for both farms considering RS and NRS

Variable	Soil type	Farm	
		*La Perla* n = 36	*Los Cedros* n = 15
pseudo-total Cd / mg kg^−1^	RS NRS	2.60 (1.80)* 0.85 (0.73)*	0.60 (0.40)* 0.16 (0.63)*
Cd-DTPA / mg kg^−1^	RS NRS	1.48 (1.97)* 0.26 (1.05)*	0.26 (0.69)* 0.03 (1.06)*
Urease / mg NH_4_^+^ kg^−1^ 2 h^−1^	RS NRS	107.41 (0.48)* 62.01 (0.40)*	72.48 (0.26)* 44.88 (0.38)*
pH	RS NRS	4.59 (0.12)* 5.00 (0.08)*	4.60 (0.07)* 4.97 (0.04)*
Ca / cmol_c_ kg^−1^	RS NRS	9.24 (0.42)* 4.17 (0.54)*	6.43 (0.36)* 4.17 (0.28)*
Mg / cmol_c_ kg^−1^	RS NRS	1.43 (0.43)* 0.53 (0.64)*	1.23 (0.45)* 0.45 (0.65)*
K / cmol_c_ kg^−1^	RS NRS	0.21 (0.27)* 0.09 (0.37)*	0.18 (0.90)* 0.09 (0.36)*
Na / cmol_c_ kg^−1^	RS NRS	0.02 (0.59)* 0.01 (0.28)*	0.04 (0.48)* 0.01 (0.29)*
EA / cmol_c_ kg^−1^	RS NRS	0.36 (1.33)* 1.82 (0.89)*	0.62 (1.03)* 4.31 (0.57)*
CEC / cmol_c_ kg^−1^	RS NRS	11.85 (0.29)* 7.52 (0.31)*	8.51 (0.28)^ns^ 8.71 (0.25)^ns^
P / mg kg^−1^	RS NRS	37.20 (0.75)* 13.25 (1.22)*	9.18 (0.56)* 1.97 (0.90)*
SOC / %	RS NRS	2.89 (0.34)* 0.65 (0.59)*	2.08 (0.39)* 0.73 (0.40)*
Zn / mg kg^−1^	RS NRS	97.51 (0.89)^ns^ 79.50 (0.60)^ns^	53.51 (0.29)^ns^ 72.08 (0.44)^ns^

* indicate a statistically significant difference (p-value < 0.05), and ns indicate a non-statistically significant difference (p-value > 0.05) according to Wilcoxon test. Cd-DTPA: Cd concentration after extraction with DTPA (considered as an approximation of plant-available Cd). Urease: urease activity. EA: Exchangeable acidity. CEC: cation exchange capacity, and SOC: soil organic carbon. The other parameters are the concentration of the elements shown and the pH values

### Cd content in rhizosphere and non-rhizosphere cacao-growing soils

**Fig. 2:**
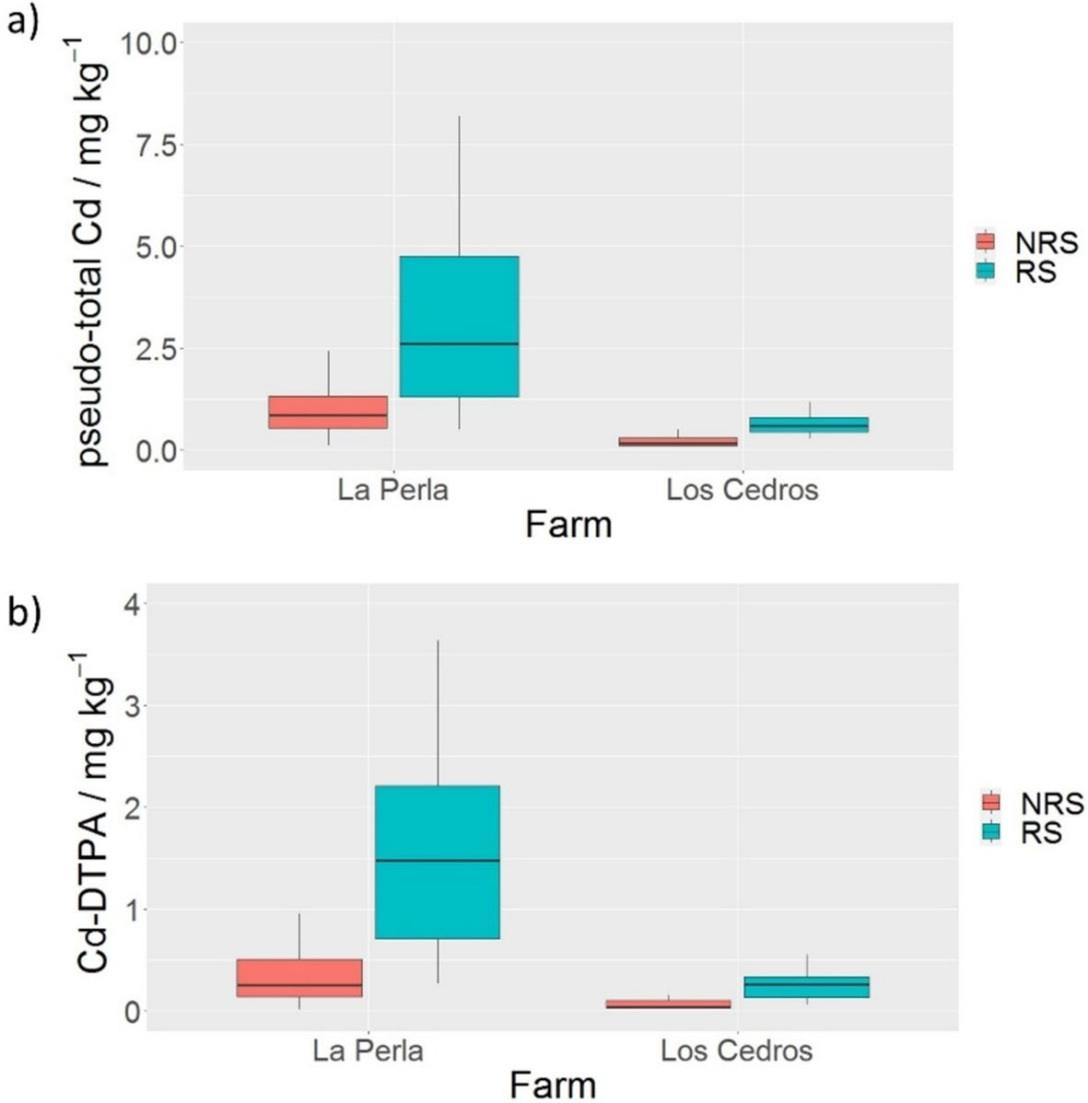
Boxplot of pseudo-total Cd (**a**) and available Cd (**b**) for both farms considering RS and NRS soils

The pseudo-total Cd global medians for RS and NRS were 1.86 and 0.57, respectively. Pseudo-total Cd was from 3.0 to 3.7 times higher in RS than in NRS considering both farms (Fig. 2a). There is no specific Cd content recommendation for cacao-growing soils (Codex Alimentarious Commission, 2022); however, an average threshold value applied to agricultural soils of 1 mg kg^−1^, based on ecological or health risks (Tóth et al., 2016), has been used to evaluate these soils (Bravo et al., 2021; Joya-Barrero et al., 2023). The sample percentages below this threshold value for *Los Cedros* farm were 93.4% for the RS and 100% for the NRS, while those from *La Perla* farm were 16.6% for the RS and 58.3% for the NRS. According to these results and the medians in Table 1, the Cd level in the farms varies depending on the soil type sampled (RS or NRS). It is worth noting that, considering all collected samples (102), 31 RS and 15 NRS were above the threshold level. Although natural cadmium concentrations in soils range from 0.01 to 1 mg kg^-1^ (Kubier et al., 2019), the higher Cd content in these soil samples could be related to the fact that the two farms are located in a geological unit overlying shales, sandstones, limestones, and cherts with possible elevated Cd levels (Gómez & Montes., 2020; Smolders & Mertens. 2013).

Pseudo-total Cd concentrations found in the NRS are in line with previous studies on the same area. A nationwide survey on Cd content in cacao-growing soils in Colombia reported, for Santander, a median pseudo-total Cd of 0.83 mg kg^−1^ (Bravo et al., 2021). Compared to other Latin American countries, the global NRS median (0.57 mg kg^−1^) is higher than those reported for Ecuador (0.33 mg kg^−1^; Argüello et al., 2019) and Honduras (0.2 mg kg^−1^ in the top-soil_0–10 cm_; Gramlich et al., 2018), and lower compared to Perú (1.7 mg kg^−1^; Scaccabarozzi et al., 2020). However, in our work, the median value of all RS (1.86 mg kg^−1^) was higher than that from the previously mentioned studies.

### Cd availability (Cd-DTPA) and its relationships with soil properties

Descriptive statistics for soil parameters showed that the data are unevenly distributed, which can be interpreted as soil conditions changing significantly from point to point, within and between farms, despite being geographically close. Consequently, there were no linear correlations between the variables; therefore, the relationships between Cd-DTPA and soil properties were evaluated by Spearman correlation coefficients (Supplementary Material Table S3). The results showed differences between variables relationships (in RS and NRS) with correlation coefficients mainly below 0.6 (p-value > 0.05). Since Spearman correlations varied significantly between the two farms, they were analyzed separately.

It is worth noting that in *La Perla* farm’s RS, pseudo-total Cd was the only variable that presented a significant correlation with Cd-DTPA (RS: ρ = 0.84, Supplementary Material Table S3), while for *Los Cedros* farm’s RS, other variables were important (e.g. SOC). This could be related to the higher Cd content found at *La Perla* farm. Higher levels of soil Cd content may exert a great influence on Cd availability (Huang et al., 2023), attenuating the effect of other soil properties. For *Los Cedros* farm’s RS, the two most important variables were SOC (ρ = 0.80) and Zn (ρ = 0.75).

Here, it must be pointed out that Cd availability to plants is complex and depends on several factors such as pH, organic matter content (Vanderschueren et al., 2021), cation exchange capacity (Hamid et al., 2019), mineral nutrition (Sarwar et al., 2010), and the presence of microorganisms (Jaramillo-Mazo et al., 2024). Furthermore, plants have been reported to influence toxic metal availability in rhizosphere soils (Seshadri et al., 2015). In this study, diethylenetriaminepentaacetic acid (DTPA) extraction was selected to assess available Cd content (Cd-DTPA), considering that DTPA is a chelating agent that mimics the interactions of soil metals with plant root exudates (Kim et al., 2015) and has been suitable for assessing soil Cd availability to cacao (Ramtahal et al., 2015). The results showed that available Cd was 5.7 to 8.6 higher in RS than in NRS (Fig. 2b), thus, the concentration of this potentially toxic element is higher in the soil near the plant's roots in both cacao farms studied. According to the correlation found between available Cd and other soil chemical properties, the variables that most influenced Cd-DTPA were pseudo-total Cd, SOC, Zn and pH, while the other variables studied were not relevant (Supplementary Material Table S3).

Soil organic matter is known to alter Cd availability (Welikala et al., 2021; Zhao et al., 2014). In this work, the measured SOC (from 1.36 to 6.03%) was like other values reported for cacao-growing soils (Cáceres et al., 2021; Gramlich et al., 2018), and all RS samples were observed to have more SOC than NRS, with a 3.9-fold difference between the global median of both soil types. The relationship between Cd-DTPA and SOC for RS (Supplementary Material Table S3), showed a strong correlation for *Los Cedros* (ρ = 0.80, p-value < 0.05), but was poor for *La Perla* farm **(**ρ = 0.26, p-value > 0.05**).** Similar results were observed for NRS.

We suggest that the differences found between the two farms in the behavior related to the SOC and Cd-DTPA relationship can be explained based on the lower Cd concentration at *Los Cedros* farm compared to *La Perla* farm (Table 1), because the contribution of SOC to Cd retention may change (Loganathan et al., 2012). Thus, the correlation between Cd-DTPA and SOC is more complex and seems to be related to the SOC (%): pseudo-total Cd (mg kg^−1^) ratio. Although the correlation was positive for both farms, it was only significant for *Los Cedros* farm with a higher ratio (Table 2). The results found for *Los Cedros* farm are in agreement with other researchers who affirm that organic matter plays an important role in soil Cd availability (Smolders & Mertens, 2013; Welikala et al., 2021; Loganathan et al., 2012). These observations help to understand why SOC may have a significant or insignificant effect on Cd availability (Li et al., 2024).

**Table 2:** Ratios SOC (%): pseudo-total Cd (mg kg^−1^)

Soil type	Farm	
	*Los Cedros*	*La Perla*
RS	3.4 SOC (%): 1 pseudo-total Cd (mg kg^−1^)	1.1 SOC (%): 1 pseudo-total Cd (mg kg^−1^)
NRS	4.5 SOC (%): 1 pseudo-total Cd (mg kg^−1^)	0.8 SOC (%): 1 pseudo-total Cd (mg kg^−1^)

Since Cd and Zn can compete for soil sorption sites (Loganathan et al., 2012), it is important to explore the correlations between them. The Zn content of the two farms studied (Table 1) is comparable to values reported elsewhere (Arévalo-Gardini et al., 2016; Argüello et al., 2019; Gramlich et al., 2017; Rodríguez-Albarracín et al., 2019). The difference in Zn content between RS and NRS was insignificant (Table 1). A positive correlation between Zn and Cd-DTPA (ρ = 0.45, p-value > 0.05 for *La Perla* farm and ρ = 0.75, p-value < 0.05 for *Los Cedros* farm) was found for RS from both farms (Supplementary Material Table S3); however, it was only significant for *Los Cedros* farm*.* A similar correlation between Zn and Cd-DTPA (ρ = 0.92) was reported in a study conducted on five cacao farms (Rodríguez-Albarracín et al., 2019). It has been pointed out that in acidic soils, Cd remains highly mobile in the presence of Zn because it reduces Cd sorption (Ming et al., 2016) and tends to form stable ionic species in the soil solution over a wide range of soil pH values (Kubier et al., 2019).

Regarding pH, we observed that 88% of RS (median pH = 4.6) was more acidic than the corresponding NRS (median pH = 5.0). A weak correlation between Cd-DTPA and pH was found for both soil types, and it was only relevant for *Los Cedros’* RS (ρ = 0.72, p-value < 0.05). These results are consistent with other studies that reported this low correlation of available Cd with pH in cacao-growing soils (Carrillo et al., 2023; Rodríguez-Albarracín et al., 2019). The high positive correlation between Cd-DTPA and pH for *Los Cedros* RS is deceiving, since it is generally accepted that increasing pH reduces the available Cd.

### Rhizosphere and non-rhizosphere soil differentiation by PCA

**Fig. 3:**
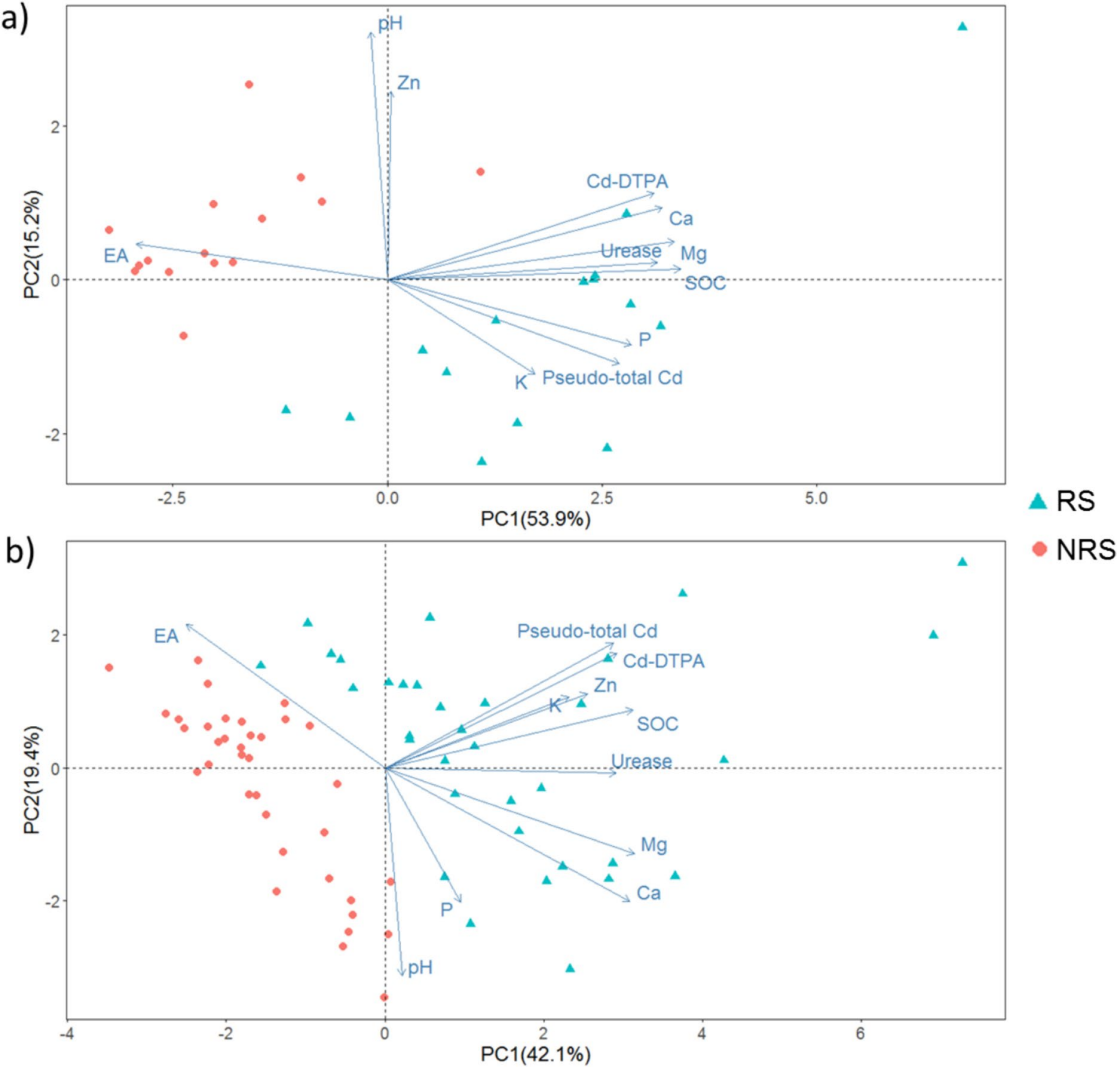
PCA Biplot for the farms **a**) *Los Cedros* and **b**)* La Perla*

Principal Component Analysis (PCA) was used to identify the variables that contribute most to the differentiation between RS and NRS, as well as to evaluate important relationships of Cd-DTPA with other soil properties (complementing the results found using classical statistics). Table S4 (Supplementary Material) shows the main results of the PCA applied to the two farms under study. The first four PCs accounted for 84.6% and 86.6% of the total variance *for La Perla* and *Los Cedros* farms, respectively. Using the first 2 PCs, the differentiation of RS and NRS is evident (Fig. 3). PCA results reveal that the most important variables for the differentiation of RS and NRS were similar when comparing *La Perla* and *Los Cedros* farms. In both farms, SOC, Ca, Mg, Cd-DTPA and urease were the main variables associated to PC1 (Supplementary Material Table S4). In relation to PC2, the results indicate the importance of pH and pseudo-total Cd. It is essential to highlight the relevance of SOC and pH because these are properties known to be affected by root activity occurring in rhizosphere soils (Richter et al., 2007). The contribution of Ca, Mg, and urease to the differentiation between RS and NRS could be related to changes on these variables by rhizosphere processes associated with plant nutrition (Dotaniya & Meena., 2015; Mcnear., 2013). It is important to mention that PCA results indicate that Cd contributes to the differentiation between both soil types.

Since variables are represented in the biplot by their correlations (Abdi & Williams., 2010), PCA revealed Cd-DTPA relationships complementary to those given by Spearman correlations analysis. At *Los Cedros* farm, Cd-DTPA had a slight positive correlation with Ca, Mg, SOC and urease and a negative correlation with EA (Fig. 3a). As for *La Perla* farm, positive correlations were observed between Cd-DTPA and pseudo-total Cd, Zn, K and SOC (Fig. 3b).

### Spatial distribution of Cd-DTPA

**Fig. 4:**
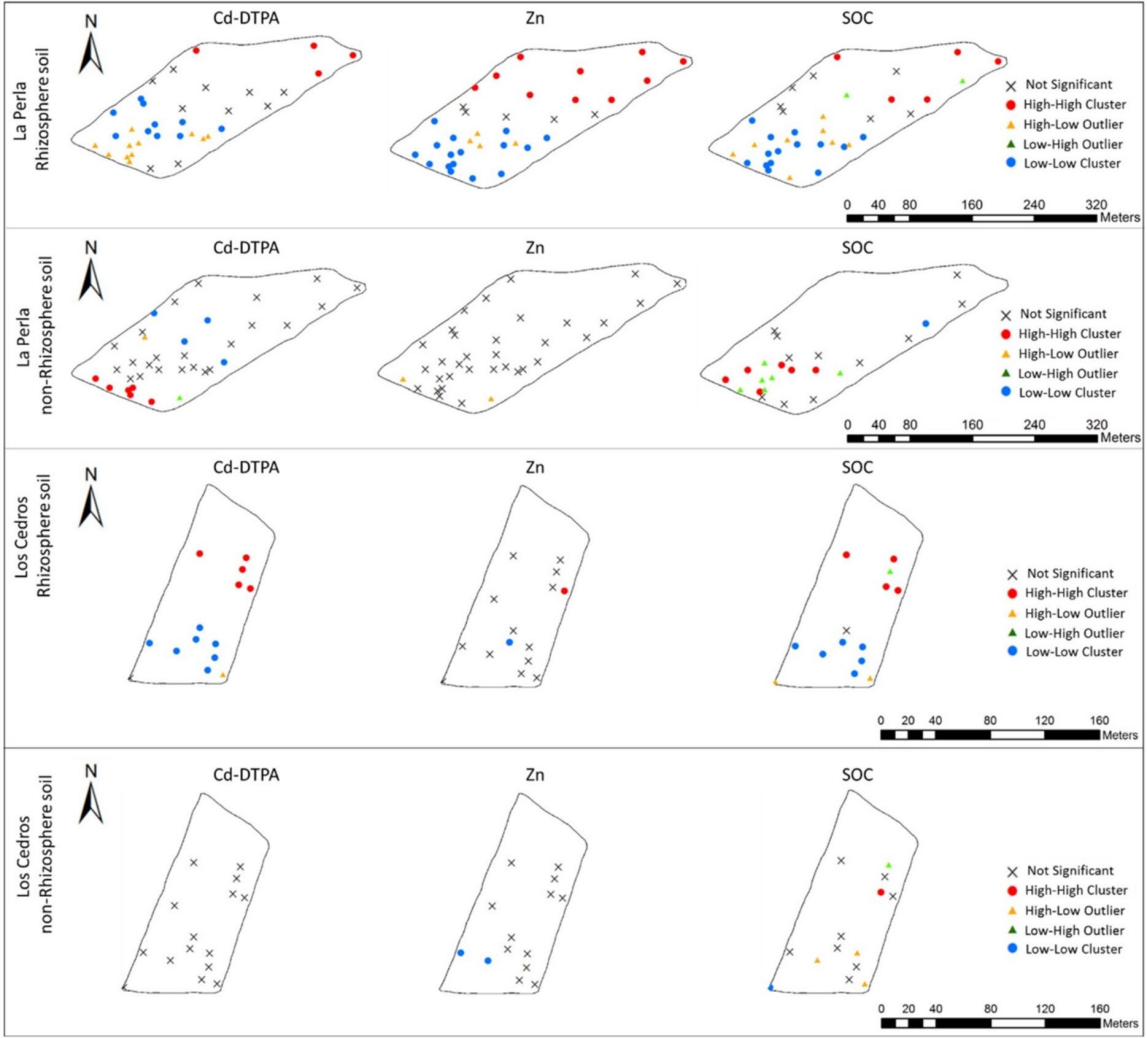
Hot and cold spots for Cd-DTPA, Zn, and SOC in the RS and NRS from *La Perla* and *Los Cedros* farms

The Global Moran's index was applied to analyze the overall spatial distribution of Cd-DTPA (Supplementary Material Table S5), followed by the Local Moran's index to identify hotspots and coldspots (Fig. 4, Supplementary Material Table S6). Since Zn and SOC showed correlation with Cd-DTPA, hotspots and coldspots from these variables were analyzed (Fig. 4). *La Perla* farm showed a similar global Moran's index for both soil types (0.2619 for RS and 0.2672 for NRS), but spatial autocorrelation was more substantial in RS according to Z-score (8.4484 for RS and 2.9614 for NRS). At *Los Cedros* farm, a clustering pattern was observed for RS, while a random distribution was found for NRS (Supplementary Material Table S5**)**. These results suggest that the soil–plant interactions in the RS have a significant influence on the spatial distribution pattern of available Cd, which was more evident at *Los Cedros* farm. RS clustering could be related to the spatial and temporal processes associated with plant rhizosphere (Hinsinger et al., 2009) and the impact of vegetation on soil metal distribution (Shi et al., 2018).

Cd-DTPA hotspots (red dots in Fig. 4) for RS at *La Perla* farm were identified at the northeast (3) and northwest (1) of the farm, while those for NRS were at the southwest (6). Cold spots (blue dots in Fig. 4) are located at the central part of *La Perla* farm for both soil types. *Los Cedros* farm’s RS has 5 hot spots, mainly by the central east area, and 7 cold spots by the south of the farm. *Los Cedros* farm’s NRS has no cold or hotspots, which has to do with the randomness of the global Moran index. The cause of the spatial distribution of hot and cold spots is not clear at this research stage; however, it is probably associated with the planted cacao trees (the soil samples were taken from different cacao trees genotypes, data not shown), since different plant genotypes may have a different impact on Cd availability in the rhizosphere (Lu et al., 2023).

The results also revealed a tendency for Cd-DTPA to be present at high concentrations in areas with high concentrations of Zn and/or SOC, only for the RS of both farms. Cd-DTPA hot spots in RS from *La Perla* farm are at the same location as Zn hot spots, and 3 of them also correspond to SOC hot spots. *Los Cedros* farm´s RS has 5 Cd-DTPA hot spots, one is consistent with a Zn hot spot and four with SOC hot spots. Another study has shown a similar spatial pattern between Cd-DTPA and SOC in wheat farm soil samples (Jafarnejadi et al., 2011). These results show that further studies focusing on geostatistical correlation analysis and other chemical properties, plant physiology, and agronomical features are needed.

## Conclusion

This study showed variations in the Cd content of both farms considering rhizosphere soils (RS) and non-rhizosphere soils (NRS). The overall results revealed that 60.8% of the RS and 29.4% of the NRS exceeded the selected threshold of 1 mg kg^−1^. Available Cd measured in cacao RS was greater than that from NRS. These findings are important considering that the plant can uptake available elements such as Cd from the rhizosphere.

The correlation analysis indicated a clear distinction between both farms. For *Los Cedros* farm, Cd-DTPA was related to different soils properties. At *La Perla* farm, it was only correlated to pseudo-total Cd. PCA results indicated that organic matter, exchangeable bases, pH and Cd are important variables to discriminate both soil types. Furthermore, PCA and hotspot analyses revealed that soil organic carbon and Zn were the main drivers of Cd presence in the cacao-growing rhizosphere soils of the studied farms.

The different chemical behavior of the studied soils and farms indicates that robust soil sampling studies will improve the understanding of Cd availability. The presence of Cd-DTPA hotspots and cold spots evidenced spatial soil variation within the farms and represents an opportunity to explore specific sites for planting cacao genotypes with high and low Cd accumulation, or for applying future remediation treatments. Inclusion of rhizosphere soils in soil sampling design will improve the diagnosis of cacao farms for Cd availability, which can be used to predict Cd concentration in cacao beans.

## Supplementary Information

Below is the link to the electronic supplementary material.

Supplementary file1 (DOCX 420 KB)

## Data Availability

No datasets were generated or analysed during the current study.
