# Supplementary material for: Cadmium availability in rhizosphere and non-rhizosphere soils in cacao farms in Santander, Colombia
Source: Environ Monit Assess. 2024 Nov 26;196(12):1254. doi: 10.1007/s10661-024-13301-x (PMC11599408; doi:10.1007/s10661-024-13301-x)
Supplement: Supplementary file 1 — Supplementary file1 (DOCX 420 KB) [file 10661_2024_13301_MOESM1_ESM.docx]

**Cadmium availability in rhizosphere and non-rhizosphere soils in cacao farms in Santander, Colombia**

#
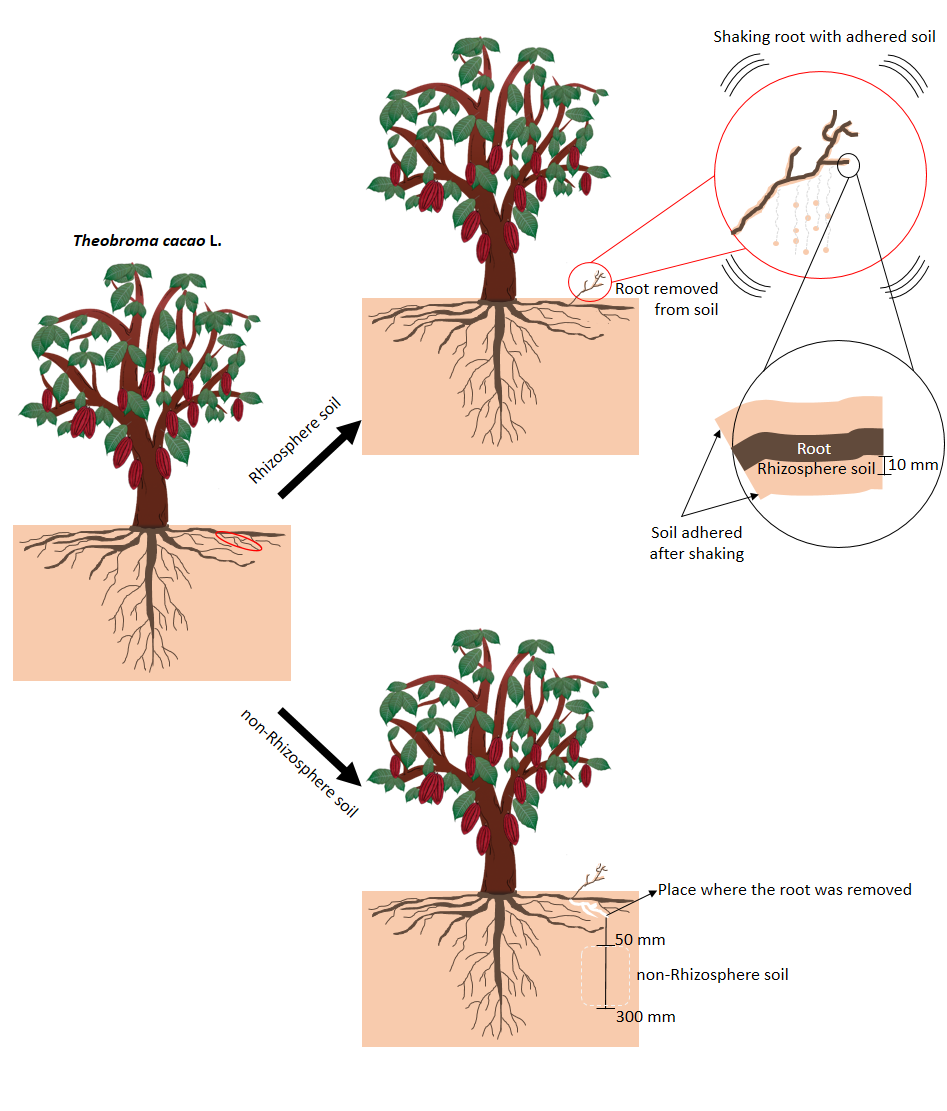


**Fig. S1.** Sampling strategy to collect rhizosphere and non-rhizosphere soils.

**Table S1.** Descriptive statistics for *La Perla* farm (n = 36).

|  |  | **RS** | | | | | **NRS** | | | | |
| --- | --- | --- | --- | --- | --- | --- | --- | --- | --- | --- | --- |
| **Variable** | **Unit** | **mean** | **sd** | **median** | **min** | **max** | **mean** | **sd** | **Median** | **min** | **max** |
| pseudo-total Cd | mg kg^-1^ | 4.70 | 5.63 | 2.60 | 0.49 | 21.29 | 1.02 | 0.75 | 0.85 | 0.11 | 3.57 |
| Cd-DTPA | mg kg^-1^ | 2.55 | 3.21 | 1.48 | 0.27 | 12.16 | 0.45 | 0.48 | 0.26 | 0.01 | 1.83 |
| urease | mg NH_4_^+^ kg^-1^ 2h^-1^ | 115.25 | 54.99 | 107.41 | 45.71 | 345.67 | 68.36 | 27.55 | 62.01 | 30.94 | 146.15 |
| pH | pH units | 4.67 | 0.54 | 4.59 | 3.83 | 5.90 | 5.16 | 0.40 | 5.00 | 4.76 | 6.32 |
| Ca | cmol_c_ kg^-1^ | 9.33 | 3.89 | 9.24 | 2.80 | 16.60 | 5.06 | 2.72 | 4.17 | 1.63 | 11.30 |
| Mg | cmol_c_ kg^-1^ | 1.55 | 0.66 | 1.43 | 0.58 | 2.90 | 0.67 | 0.43 | 0.53 | 0.13 | 1.70 |
| K | cmol_c_ kg^-1^ | 0.22 | 0.06 | 0.21 | 0.16 | 0.48 | 0.11 | 0.04 | 0.09 | 0.06 | 0.25 |
| Na | cmol_c_ kg^-1^ | 0.02 | 0.01 | 0.02 | 0.01 | 0.06 | 0.01 | 0.00 | 0.01 | 0.01 | 0.01 |
| EA | cmol_c_ kg^-1^ | 0.99 | 1.32 | 0.36 | 0.01 | 4.70 | 2.20 | 1.97 | 1.82 | 0.01 | 7.26 |
| CEC | cmol_c_ kg^-1^ | 12.11 | 3.54 | 11.85 | 5.44 | 18.80 | 8.04 | 2.47 | 7.52 | 4.23 | 12.70 |
| P | mg kg^-1^ | 41.10 | 30.96 | 37.20 | 3.88 | 116.00 | 33.88 | 41.29 | 13.25 | 0.36 | 116.00 |
| SOC | % | 3.15 | 1.06 | 2.89 | 1.63 | 6.03 | 0.60 | 0.35 | 0.65 | 0.15 | 1.41 |
| Zn | mg kg^-1^ | 132.89 | 118.88 | 97.51 | 34.93 | 582.87 | 93.62 | 56.42 | 79.50 | 31.02 | 305.21 |

Cd-DTPA: Cd concentration after extraction with DTPA (considered as an approximation to the available Cd to the plant). Urease: urease activity. EA: exchangeable acidity. CEC: cation exchange capacity. and SOC: soil organic carbon. The other parameters are the concentration of the elements and the pH.

**Table S2.** Descriptive statistics for *Los Cedros* farm (n = 15).

|  |  | **RS** | | | | | **NRS** | | | | |
| --- | --- | --- | --- | --- | --- | --- | --- | --- | --- | --- | --- |
| **Variable** | **Unit** | **mean** | **sd** | **median** | **min** | **max** | **mean** | **sd** | **median** | **min** | **max** |
| pseudo-total Cd | mg kg^-1^ | 0.61 | 0.25 | 0.60 | 0.27 | 1.16 | 0.21 | 0.13 | 0.16 | 0.08 | 0.49 |
| Cd-DTPA | mg kg^-1^ | 0.26 | 0.18 | 0.26 | 0.06 | 0.72 | 0.08 | 0.08 | 0.03 | 0.02 | 0.24 |
| urease | mg NH_4_^+^ kg^-1^ 2h^-1^ | 75.45 | 19.48 | 72.48 | 52.33 | 123.11 | 43.58 | 16.45 | 44.88 | 11.08 | 81.44 |
| pH | pH units | 4.66 | 0.33 | 4.60 | 4.28 | 5.55 | 5.01 | 0.18 | 4.97 | 4.76 | 5.36 |
| Ca | cmol_c_ kg^-1^ | 6.94 | 2.49 | 6.43 | 3.45 | 12.40 | 3.99 | 1.13 | 4.17 | 1.94 | 5.83 |
| Mg | cmol_c_ kg^-1^ | 1.32 | 0.60 | 1.23 | 0.45 | 2.81 | 0.55 | 0.36 | 0.45 | 0.11 | 1.38 |
| K | cmol_c_ kg^-1^ | 0.26 | 0.23 | 0.18 | 0.11 | 1.05 | 0.10 | 0.04 | 0.09 | 0.05 | 0.19 |
| Na | cmol_c_ kg^-1^ | 0.05 | 0.02 | 0.04 | 0.02 | 0.09 | 0.01 | 0.00 | 0.01 | 0.01 | 0.02 |
| EA | cmol_c_ kg^-1^ | 0.88 | 0.91 | 0.62 | 0.01 | 3.31 | 4.18 | 2.38 | 4.31 | 0.23 | 8.15 |
| CEC | cmol_c_ kg^-1^ | 9.46 | 2.62 | 8.51 | 6.11 | 15.50 | 8.84 | 2.21 | 8.71 | 5.65 | 12.70 |
| P | mg kg^-1^ | 10.19 | 5.70 | 9.18 | 3.24 | 25.30 | 2.83 | 2.56 | 1.97 | 0.36 | 10.00 |
| SOC | % | 2.27 | 0.88 | 2.08 | 1.36 | 4.87 | 0.78 | 0.31 | 0.73 | 0.43 | 1.70 |
| Zn | mg kg^-1^ | 57.37 | 16.79 | 53.51 | 32.05 | 96.41 | 73.50 | 32.28 | 72.08 | 26.47 | 165.72 |

Cd-DTPA: Cd concentration after extraction with DTPA (considered as an approximation to the available Cd to the plant). Urease: urease activity. EA: exchangeable acidity. CEC: cation exchange capacity. and SOC: soil organic carbon. The other parameters are the concentration of the elements and the pH.

**Table S3**. Spearman correlation coefficients between Cd-DTPA and the other 12 chemical properties measured in this work, considering RS and NRS for both farms.

|  | ***La Perla* farm** | | ***Los Cedros* farm** | |
| --- | --- | --- | --- | --- |
| **Variable** | **RS**  **Cd-DTPA** | **NRS**  **Cd-DTPA** | **RS**  **Cd-DTPA** | **NRS**  **Cd-DTPA** |
| pseudo-total Cd | 0.84* | 0.95* | *0.43* | *0.34* |
| CEC | *0.09* | -0.65* | *0.56* | *-0.39* |
| Urease | *0.42* | *-0.32* | *0.48* | *-0.01* |
| Zn | *0.45* | *-0.26* | 0.75* | *0.14* |
| Ca | *0.21* | *-0.44* | 0.61* | *0.46* |
| Na | *-0.05* | *-0.43* | *0.37* | *0.30* |
| SOC | *0.26* | *0.30* | 0.80* | 0.64* |
| EA | *-0.30* | *-0.15* | -0.77* | *-0.55* |
| pH | *0.22* | *-0.06* | 0.72* | *0.44* |
| K | *0.21* | *-0.05* | *0.22* | *0.03* |
| P | *0.02* | *0.20* | *0.20* | *0.15* |
| Mg | *0.06* | *-0.11* | *0.58* | *0.21* |

Spearman correlation: * indicate a statistically significant correlation (p-value < 0.05) and italics values indicate a non-statistically significant correlation (p-value > 0.05).

**Table S4**. Loadings of variables to PCs for the two farms investigated in this work.

|  | ***La Perla* farm** | | | |  | ***Los Cedros* farm** | | | |
| --- | --- | --- | --- | --- | --- | --- | --- | --- | --- |
| **Variable** | **PC1** | **PC2** | **PC3** | **PC4** | **Variable** | **PC1** | **PC2** | **PC3** | **PC4** |
| Mg | **0.366** | -0.222 | 0.217 | 0.155 | SOC | **0.382** | 0.030 | -0.052 | 0.152 |
| SOC | **0.364** | 0.150 | 0.364 | 0.046 | Mg | **0.375** | 0.105 | 0.079 | -0.176 |
| Ca | **0.360** | -0.345 | 0.112 | 0.158 | Ca | **0.359** | 0.197 | 0.117 | -0.086 |
| Cd-DTPA | **0.341** | 0.297 | -0.359 | -0.089 | Urease | **0.352** | 0.048 | -0.272 | -0.222 |
| Urease | **0.340** | -0.014 | 0.056 | 0.458 | Cd-DTPA | **0.348** | 0.237 | -0.049 | 0.337 |
| Pseudo-total Cd | **0.335** | **0.322** | -0.334 | -0.178 | P | **0.319** | -0.179 | 0.152 | -0.183 |
| Zn | 0.297 | 0.192 | -0.439 | 0.044 | Pseudo-total Cd | **0.303** | -0.230 | 0.028 | 0.591 |
| K | 0.270 | 0.184 | 0.470 | -0.309 | K | 0.193 | -0.259 | 0.589 | -0.464 |
| P | 0.111 | -0.347 | -0.051 | -0.737 | Zn | 0.004 | 0.517 | 0.591 | 0.268 |
| pH | 0.025 | -0.538 | -0.380 | 0.179 | pH | -0.022 | **0.680** | -0.234 | -0.302 |
| EA | -0.292 | 0.370 | 0.062 | 0.165 | EA | -0.328 | 0.097 | 0.356 | 0.121 |
| Variance explain (%) | 42.1 | 19.4 | 13.9 | 9.2 | Variance explain (%) | 53.9 | 15.2 | 10.8 | 6.7 |

Bold values indicate the variables mainly associated to PC1 and PC2.

**Table S5.** Global Moran’s index and spatial pattern for Cd-DTPA.

| **Farm** | **Soil type** | **Global Moran’s index** | **z-score** | **p-value** | **Spatial pattern** |
| --- | --- | --- | --- | --- | --- |
| *La Perla* | RS | 0.2619 | 8.4484 | <1.0x10^-6^ | Clustered |
|  | NRS | 0.2672 | 2.9614 | 3.1x10^-3^ | Clustered |
| *Los Cedros* | RS | 0.5509 | 5.0453 | 1.0x10^-6^ | Clustered |
|  | NRS | -0.1873 | -1.4849 | 0.1377 | Random |

**Table S6**. Sample percent of local spatial pattern types.

| **Farm** | **Soil Type** | **High-High** | **Low-Low** | **High-Low** | **Low-High** | **Not significant** |
| --- | --- | --- | --- | --- | --- | --- |
| *La Perla* | RS | 11.4 | 28.6 | 31.4 | 0 | 28.6 |
| *La Perla* | NRS | 17.1 | 11.4 | 2.9 | 2.9 | 65.7 |
| *Los Cedros* | RS | 35.7 | 50.0 | 7.15 | 0 | 7.15 |
| *Los Cedros* | NRS | 0 | 0 | 0 | 0 | 100 |
